# Opsoclonus-myoclonus syndrome associated with a nasopharyngeal tumor in an adult: a case report

**DOI:** 10.1186/s13256-015-0605-9

**Published:** 2015-06-02

**Authors:** Bilal Gani Taib, Andrew J Kinshuck, Philip Milburn-McNulty, Lauren Fratalia, Leigh Forsyth, David Husband, Terry M Jones, Anu Jacob

**Affiliations:** 1 Royal Liverpool Hospital University Hospital, Postgraduate Office, Prescot Street, Liverpool, L7 8XP UK; 2 Department of Otolaryngology, Head and Neck Surgery, University Hospital Aintree, Longmoor Lane, Liverpool, L9 7AL UK; 3 Department of Neurology, Walton Centre NHS Foundation Trust, Lower Lane, Liverpool, L9 7LJ UK; 4 Department of Cellular Pathology, St Helens & Knowsley NHS Trust, Marshalls Cross Road, Merseyside, WA9 3DA UK; 5 Clatterbridge Cancer Centre NHS Foundation Trust, Clatterbridge Road, Wirral, Merseyside, CH63 4JY UK; 6 Liverpool CR-UK Centre, University of Liverpool, Daulby Street, Liverpool, L69 3GA UK

**Keywords:** Oncology, Paraneoplastic, Ataxia

## Abstract

**Introduction:**

Opsoclonus-myoclonus syndrome is a rare autoimmune syndrome usually seen in children and very rarely in adults. It typically presents with a triad of opsoclonus, myoclonus and ataxia, and is most often associated with a tumor or after an infection or vaccination. Around half of all adult cases are paraneoplastic in origin, and isolated case reports include associations with lung, breast and ovarian cancers. To the best of our knowledge, this is the first-ever reported case of paraneoplastic opsoclonus-myoclonus syndrome occurring in association with a nasopharyngeal carcinoma.

**Case presentation:**

A 50-year-old British Caucasian woman presented with left-sided otalgia and subjective hearing loss. Over the coming weeks she developed subacute confusion and dizziness, leading to recurrent falls. Her clinical examination revealed opsoclonus, myoclonus and signs of cerebellar dysfunction. Subsequent magnetic resonance imaging revealed a left-sided nasopharyngeal carcinoma, which was confirmed on biopsy. A tapering dose of steroids and a five-day course of intravenous immunoglobulins, followed by a combination of chemo-radiotherapy for the nasopharyngeal carcinoma, led to a significant clinical improvement. At six months follow-up she had no signs of focal neurological deficit, apart from the inability to tandem walk. We believe that the typical clinical features, presence of a tumor and response to treatment support a paraneoplastic aetiology.

**Conclusions:**

We show that a nasopharyngeal carcinoma can be associated with adult onset opsoclonus-myoclonus syndrome. Both neurologists and otorhinolaryngologists must be aware of such a presentation. Prognosis of the syndrome depends on early and adequate management of the tumor, therefore prompt identification of the syndrome and the underlying tumor is essential.

## Introduction

Opsoclonus-myoclonus syndrome (OMS) is known by a variety of names including, ‘dancing eye-dancing limb syndrome’, opsoclonus-myoclonus-ataxia and Kinsbourne syndrome, following the first description in an infant by Marcel Kinsbourne in 1962 [1,2]. The variable nomenclature for OMS reflects how patients can lack any one of the classical triad of opsoclonus, myoclonus and ataxia [3]. OMS typically presents in children between the ages of one to four-years-old, where it is most often associated with a paraneoplastic, immune-mediated encephalopathy as a result of a neuroblastoma [4]. An identical presentation may occur without a tumor when it typically occurs after infections or vaccinations. It is less common in adults, where up to 50% are thought to be paraneoplastic in origin [1]. The most commonly associated tumors are non-small cell and small cell lung carcinomas, breast cancers and ovarian cancers [5,6]. To the best of our knowledge, this is the first reported case of paraneoplastic OMS occurring in association with a nasopharyngeal carcinoma.

## Case presentation

A 50-year-old British Caucasian woman presented to her general practitioner with a six-month history of pain in and around the left mastoid process and subjective mild hearing loss. She was referred to the local district general hospital where an ear, nose and throat exam with a pure tone audiogram was normal, except for tenderness over the left temporomandibular joint. Whilst awaiting a head and neck magnetic resonance imaging scan (MRI) she presented to her local hospital with subacute onset dizziness, confusion and gait instability leading to recurrent falls. She was subsequently admitted to the regional tertiary neurological centre. On examination, she was confused and disorientated, with a Mini Mental State Examination score of 19 out of 30. She had oscillopsia associated with bilateral, conjugate, involuntary, random eye movements consistent with opsoclonus, mild dysarthria, titubation, upper limb myoclonus, left arm dysmetria and a coarse intention tremor. She was not able to sit upright due to a severe truncal ataxia. Her general and systemic examination was normal. A putative diagnosis of OMS was made, and further investigations were arranged in order to further investigate the cause.

Her full blood counts, C-reactive protein, liver and renal function tests were normal. Blood cultures taken to identify an infective cause were negative. A paraneoplastic antibody screen was negative for the following antibodies: anti-neuronal nuclear antibodies type 1, anti- Hu, Purkinje cell antibodies, anti-neuronal nuclear antibodies type II, anti-Ma, anti-Tr, anti-amphiphysin, collapsin response mediator protein-5, sex determining region Y-box 1 antibodies, N-methyl D-aspartate antibodies, voltage gated potassium channel antibodies, double-stranded DNA antibodies, extractable nuclear antigen antibodies and anti-neutrophil cytoplasmic antibodies. Tumor markers, including cancer antigens 19–9 and 125, carcinoembryonic antigen, alpha feta protein and human chorionic gonadotropin, were also all unremarkable. A traumatic lumbar puncture tap contained a raised protein level of 0.71 and occasional mononuclear cells. However, no organisms were detected.

**Figure 1:**
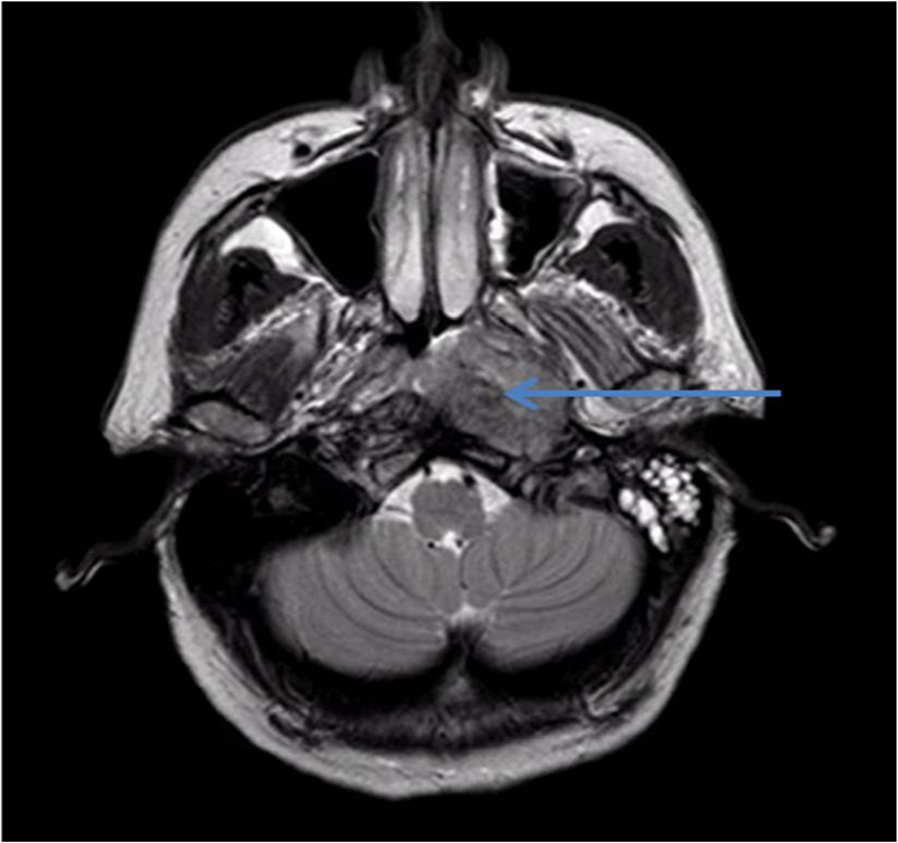
T2-weighted axial magnetic resonance image depicting a left-sided nasopharyngeal mass (arrow) and fluid filling left mastoid air cells.

**Figure 2:**
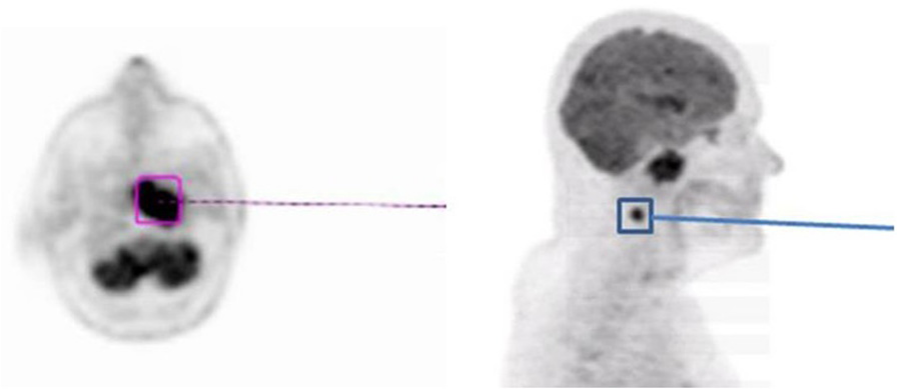
Whole body (18F)-fluorodeoxyglucose positron emission tomography depicting a mass in the post-nasal space (purple box) and an ipsilateral hypermetabolic level II lymph node (1.5×1.4cm) (blue box).

An MRI scan of her brain revealed a mass in the left nasopharynx (3.4×2.5×2.6cm) with possible bony erosion of the skull base. There was fluid collection in the left mastoid air cells (Figure 1) and local mucosal enhancement consistent with inflammatory disease. Subsequent whole body (18F)-fluorodeoxyglucose positron emission tomography (PET) did not reveal any other tumors, but confirmed the presence of a nasopharyngeal mass and an ipsilateral hypermetabolic level II lymph node (1.5×1.4cm) (Figure 2).

**Figure 3:**
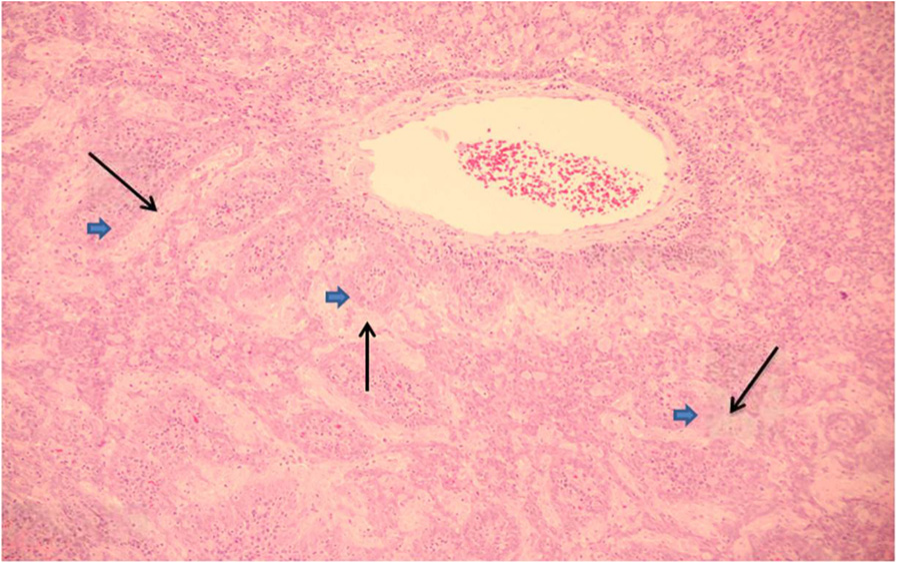
Representative area of the nasopharyngeal carcinoma with interconnecting cords of paler epithelial cells (arrows) surrounded by an outer layer of smaller ‘basaloid’ (arrow heads) cells. (×10 magnification, hematoxylin and eosin stain).

**Figure 4:**
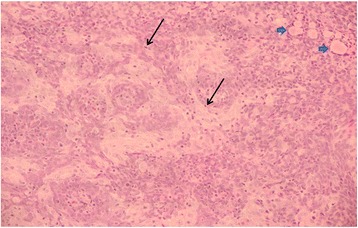
Representative area of nasopharyngeal carcinoma at x20 magnification. The strands of malignant cells can be seen more clearly. In addition to the pale cords and the darker basaloid cells, scattered lymphocytes can be seen (the small dark cells scattered throughout the image, arrows) and deposits of amyloid-like protein (the pink globules forming a band at the top right of the image, arrowheads). (×20 magnification, hematoxylin and eosin stain).

Following identification of the mass, rigid nasoendoscopy under general anaesthetic was performed, and showed a pale bulky lesion in the post-nasal space blocking the left Eustachian tube. This was biopsied, and histological findings showed fragments of a neoplasm, consisting of interconnecting cords of cells with a biphasic pattern (Figure 3). The outer layer had a basaloid, spindled appearance, whilst the inner cell clusters had more abundant, eosinophilic, squamoid cytoplasm. Globules of pink amyloid-like protein were present in both cell groups. The intervening stroma contained a lymphoplasmacytic stromal infiltrate, which also extended into the epithelium (Figure 4). Immunocytochemistry showed positivity for MNF-116 and CAM5.2 (antibodies against epitopes expressed by different cytokeratins within epithelial cells); the latter also appeared to stain the pink globules. The p63 antibody (a marker of squamous differentiation expressed in basal and parabasal nuclei of nasopharyngeal epithelium) predominantly stained the outer basaloid layers whilst the S100 protein (a marker present in neural, melanocytic and antigen presenting cells) highlighted scattered dendritic cells. These findings were characteristic of a nasopharyngeal carcinoma.

She was treated with a three-day course of 1g methylprednisolone (Pfizer Limited, Tadworth, Surrey, UK) administered intravenously, followed by a tapering 60mg dose of oral prednisolone (Actavis, Barnstaple, Devon, UK) for several weeks for her neurological symptoms. She also received five days of intravenous immunoglobulins (IVIG) at a dose of 0.4g/kg/day (Biotest Pharma GmbH, Landsteinerstr. 5 Dreieich, Germany). Her involuntary movements and ataxia improved considerably, and in a few weeks she was able to walk with a cane. But after one month of receiving steroid treatment she developed a psychotic episode, presumably related to the steroids, that promptly responded to a reduction in steroid doses and haloperidol. Following a referral to oncology she was treated with chemoradiation: 70Gy in 35 fractions over seven weeks, using intensely modulated radiotherapy delivered by VMAC (Volumetric Arc Therapy, RapidArcTM, Varian Medical Systems, Palo Alto, CA, USA) and three cycles of concurrent cisplatin chemotherapy at a dose of 80mg/m^2^ per cycle. Four months from her diagnosis she was able to sit upright without support. She still had rare fragments of opsoclonus, which were asymptomatic. At six months follow-up there were no signs of focal neurological deficit, apart from the inability to tandem walk.

## Discussion

The differential diagnosis of myoclonus and ataxia are many, and include progressive cerebellar ataxia and myoclonus syndromes. However, the presence of opsoclonus distinguishes the syndrome as OMS. To the best of our knowledge, this is the first reported case of paraneoplastic OMS occurring in association with a nasopharyngeal carcinoma. Although no paraneoplastic anti-neuronal antibody was found, we believe that the presence of a typical paraneoplastic syndrome in association with a tumor and an excellent resolution of symptoms are sufficient for the diagnosis. This view is reinforced by Graus *et al*., who defined the diagnostic criteria for paraneoplastic syndromes [7]. Musunuru and Kesari [6] reported initial seronegativity followed by seropositivity, leading us to speculate that an as yet undescribed antibody may be responsible for OMS.

Childhood OMS exhibits a monophasic or chronic relapsing pattern. It rarely regresses spontaneously, and removal of a tumor, if present, is only sometimes curative [8]. The current standard in children is the use of tapered corticosteroids at 2mg/kg/day, or adrenocorticotropic hormone (ACTH) [3]. IVIG and rituximab have been tried with variable success. A combination of ACTH, IVIG and rituximab, an example of multimodal immunosuppression, have led to relapse rates of 17% over six months, which is much lower than for conventional agents alone [9]. Further research is required on the treatment of OMS, and two clinical trials exploring the use of steroids with rituximab or cyclophosphamide and tacrolimus are recruiting at present [10,11].

## Conclusions

In this case report we highlight a hitherto unknown presentation of a nasopharyngeal cancer as OMS. Small nasopharyngeal lesions could be overlooked on brain imaging scans, and will not be picked up on routine computed tomography (CT) thorax, abdomen and pelvis scans used to try to identify a paraneoplastic cause for OMS. Both neurologists and otorhinolaryngologists must be aware of such a presentation. Where needed, careful search for a tumor with a PET imaging scan should be considered in order to identify cancers at a treatable stage, as in our patient.

## Consent

Written informed consent was obtained from the patient for publication of this case report and accompanying images. A copy of the written consent is available for review by the Editor-in-Chief of this journal.

## Abbreviations

ACTH: Adrenocorticotropic hormone
IVIG: Intravenous immunoglobulin
MRI: Magnetic resonance imaging
OMS: Opsoclonus-myoclonus syndrome
PET: Positron emission tomography
